# Dichloridobis(thio­urea-κ*S*)nickel(II)

**DOI:** 10.1107/S1600536812006174

**Published:** 2012-02-24

**Authors:** Hafid Zouihri

**Affiliations:** a Laboratoire Privé de Cristallographie (LPC), Kénitra, Morocco

## Abstract

The title complex, [NiCl_2_(CH_4_N_2_S)_2_], has been synthesized from the previously reported (diamino­methyl­idene)sulfonium chloride–thio­urea (3/2) salt [Zouihri (2012*b* ▶). *Acta Cryst.* E**68**, o257]. The Ni^II^ ion is coordinated in a distorted tetra­hedral geometry by two mol­ecules of thio­urea [Ni—S = 2.3079 (7) and 2.3177 (6) Å] and two chloride anions [Ni—Cl = 2.2516 (7) and 2.2726 (7) Å]. The bond angles at the Ni atom lie between 96.69 (2) and 115.40 (3)°, while the dihedral angle between the mean planes of the two thio­urea ligands is 6.36 (15)°. The crystal structure is characterized by intra- and inter­molecular N—H⋯Cl hydrogen bonds, which lead to the formation of two-dimensional networks lying parallel to the *ab* plane. The networks are linked *via* classical N—H⋯Cl and N—H⋯S hydrogen bonds, forming a three-dimensional arrangement.

## Related literature

For the synthesis and the crystal structure of (diamino­methyl­idene)sulfonium chloride thio­urea (3/2), see: Zouihri (2012*b* ▶). For related structures, see: Ambujam *et al.* (2007 ▶); Zouihri (2012*a* ▶). For related literature on the coordination complexes of Ni^II^ salts with thio­urea, see: Asif *et al.* (2010 ▶).
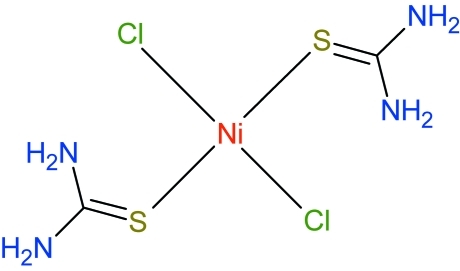


## Experimental

### 

#### Crystal data

[NiCl_2_(CH_4_N_2_S)_2_]
*M* *_r_* = 281.85
Monoclinic, 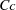

*a* = 8.1578 (3) Å
*b* = 11.8183 (5) Å
*c* = 10.8526 (6) Å
β = 103.869 (2)°
*V* = 1015.81 (8) Å^3^
*Z* = 4
Mo *K*α radiation
μ = 2.79 mm^−1^
*T* = 100 K
0.42 × 0.37 × 0.17 mm

#### Data collection

Bruker APEXII CCD detector diffractometer
Absorption correction: multi-scan (*SADABS*; Sheldrick, 2004 ▶) *T*_min_ = 0.322, *T*_max_ = 0.622
4883 measured reflections
1695 independent reflections
1678 reflections with *I* > 2σ(*I*)
*R*_int_ = 0.022

#### Refinement

*R*[*F* ^2^ > 2σ(*F* ^2^)] = 0.017
*wR*(*F* ^2^) = 0.045
*S* = 1.08
1695 reflections
132 parameters
10 restraints
All H-atom parameters refined
Δρ_max_ = 0.25 e Å^−3^
Δρ_min_ = −0.15 e Å^−3^
Absolute structure: Flack (1983 ▶), 745 Friedel pairs
Flack parameter: 0.069 (10)

### 

Data collection: *APEX2* (Bruker, 2005 ▶); cell refinement: *SAINT* (Bruker, 2005 ▶); data reduction: *SAINT*; program(s) used to solve structure: *SHELXS97* (Sheldrick, 2008 ▶); program(s) used to refine structure: *SHELXL97* (Sheldrick, 2008 ▶); molecular graphics: *PLATON* (Spek, 2009 ▶); software used to prepare material for publication: *publCIF* (Westrip, 2010 ▶).

## Supplementary Material

Crystal structure: contains datablock(s) I, global. DOI: 10.1107/S1600536812006174/fj2518sup1.cif

Structure factors: contains datablock(s) I. DOI: 10.1107/S1600536812006174/fj2518Isup2.hkl

Additional supplementary materials:  crystallographic information; 3D view; checkCIF report

## Figures and Tables

**Table 1:** Hydrogen-bond geometry (Å, °)

*D*—H⋯*A*	*D*—H	H⋯*A*	*D*⋯*A*	*D*—H⋯*A*
N1—H1*A*⋯Cl1	0.84 (3)	2.60 (3)	3.388 (3)	157 (3)
N1—H1*B*⋯Cl2^i^	0.83 (3)	2.56 (3)	3.365 (3)	164 (3)
N2—H2*A*⋯Cl2^i^	0.83 (3)	2.75 (3)	3.499 (2)	150 (3)
N2—H2*B*⋯Cl2^ii^	0.81 (2)	2.64 (2)	3.432 (2)	166 (3)
N3—H3*A*⋯Cl1^iii^	0.86 (3)	2.83 (5)	3.423 (3)	128 (5)
N3—H3*B*⋯Cl2^iv^	0.86 (4)	2.47 (4)	3.317 (3)	168 (4)
N4—H4*A*⋯S2^v^	0.84 (3)	2.70 (3)	3.366 (2)	137 (3)
N4—H4*B*⋯Cl1	0.86 (3)	2.60 (3)	3.448 (3)	168 (3)

Symmetry codes: (i) 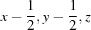; (ii) 
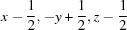
; (iii) 
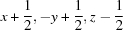
; (iv) 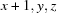; (v) 
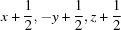
.

**Fig. 1.**
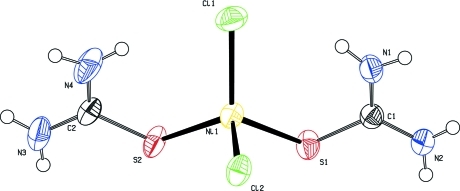
Molecular view of the title compound showing displacement ellipsoids drawn at the 50% probability level.

**Fig. 2.**
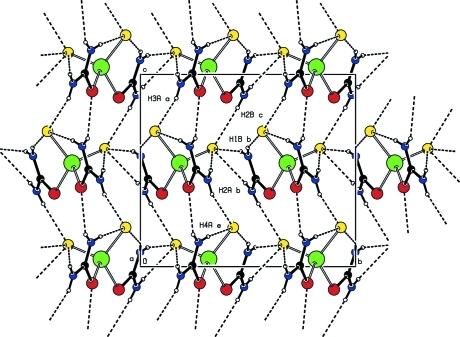
Projection of the title compound along the a axis showing two-dimensional networks lying parallel to the ab plane, H-bonds are represented by dashed lines.

**Fig. 3.**
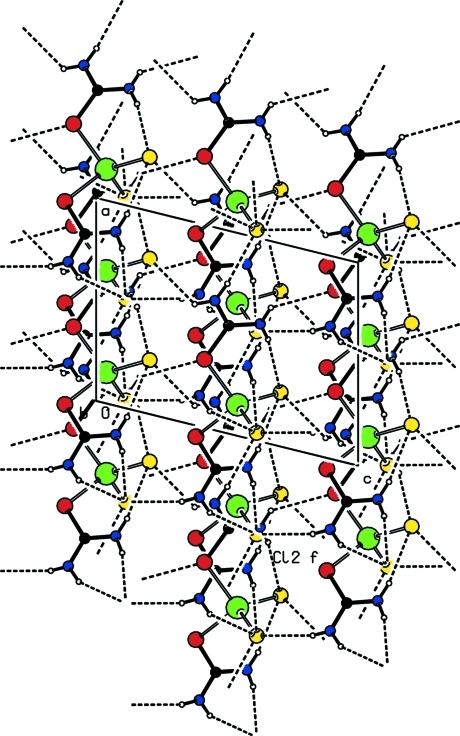
Projection of the title compound along the b axis showing the three-dimensional arrangement of the title complexe, H-bonds are represented by dashed lines.

## supplementary crystallographic information

### Comment

Nickel^(II)^, which has a d^8^ configuration, commonly exhibits octahedral, square planar and tetrahedral coordination geometries depending upon the nature of the ligands and the Crystal Field Splitting Parameter value.

In our case, the coordination complexes of Ni^(II)^ salts with thiourea show a variety of compositions and types of coordination (octahedral, tetragonal, square-planar and tetrahedral) (Asif *et al.* 2010). In general, the predominant coordination geometries for the Ni^(II)^-Ligand-*X* (*X*= Cl^-^, Br^-^ and I^-^) are Tetragonal (Ni^(II)^*L*_4_)*X*_2_ and Octahedral (Ni^(II)^*L*_6_)*X*_2_. Tetrakis coordiantion of thiourea about Nickel Ni(Th)_4_Cl_2_ has been found in centered tetragonal symmetry class I4 by K. Ambujam (Ambujam *et al.* 2007).

In former work we have reported the synthesis and crystal structure of the *catena*-poly[[chlorido(thiourea-κ*S*)copper(I)]-µ-thiourea-κ^2^*S*:*S*] complexe (Zouihri, 2012*a*). In this paper we report the crystal structure of [Ni^(II)^(Th)_2_] 2Cl^-^ which has been synthetized from the (Diaminomethylidene)sulfonium chloride-thiourea (3/2) (Zouihri, 2012*b*).

In the title complexe compound, (SCN_2_H_4_)_2_Ni^(II)^Cl_2_, The Ni^(II)^ atom is four coordinated in a tetrahedral geometry by two molecules of thiourea (average Ni—S distance = 2.3079 (7) to 2.3177 (6) Å) and two chloride anions (average Ni—Cl distance = 2.2516 (7) to 2.2726 (7) Å) with average (S, Cl)—Ni^II^—(S, Cl) torsion angles between 96.69 (2)° and 115.40 (3)°. The dihedral angle between the two thiourea Ligands is: 6.36 (15)°.

The crystal structure is characterized by intramolecular and intermolecular N—H···Cl hydrogen bonds which lead to the formation of two-dimensional networks lying parallel to the *ab* plane (Fig. 2 and Table 1). The networks are linked *via* classical intermolecular N—H···Cl and N—H···S hydrogen bonds, forming a three-dimensional arrangement (Fig. 3 and Table 1).

### Experimental

To a 10 ml aqueous solution of NiCl2 (2 mmol) was added 10 ml EtOH solution of (Diaminomethylidene)sulfonium chloride-thiourea (3/2) (Zouihri, 2012*b*) (1.0 mmol). Colourless crystal were obtained after about one week.

### Refinement

All H atoms were located from difference Fourier maps and refined isotropically, with restained distance N—H = 0.86 (2) A.

### Crystal data

**Table:**

[NiCl_2_(CH_4_N_2_S)_2_]	*F*(000) = 568
*M**_r_* = 281.85	*D*_x_ = 1.843 Mg m^−^^3^
Monoclinic, *C**c*	Mo *K*α radiation, λ = 0.71073 Å
Hall symbol: C -2yc	Cell parameters from 289 reflections
*a* = 8.1578 (3) Å	θ = 1.8–26.7°
*b* = 11.8183 (5) Å	µ = 2.79 mm^−^^1^
*c* = 10.8526 (6) Å	*T* = 100 K
β = 103.869 (2)°	Prism, colourless
*V* = 1015.81 (8) Å^3^	0.42 × 0.37 × 0.17 mm
*Z* = 4	

### Data collection

**Table:**

Bruker APEXII CCD detector diffractometer	1695 independent reflections
Radiation source: fine-focus sealed tube	1678 reflections with *I* > 2σ(*I*)
Graphite monochromator	*R*_int_ = 0.022
ω and φ scans	θ_max_ = 25.5°, θ_min_ = 3.1°
Absorption correction: multi-scan (*SADABS*; Sheldrick, 2004)	*h* = −9→8
*T*_min_ = 0.322, *T*_max_ = 0.622	*k* = −14→14
4883 measured reflections	*l* = −13→13

### Refinement

**Table:**

Refinement on *F*^2^	Secondary atom site location: difference Fourier map
Least-squares matrix: full	Hydrogen site location: inferred from neighbouring sites
*R*[*F*^2^ > 2σ(*F*^2^)] = 0.017	All H-atom parameters refined
*wR*(*F*^2^) = 0.045	*w* = 1/[σ^2^(*F*_o_^2^) + (0.0205*P*)^2^ + 0.1021*P*] where *P* = (*F*_o_^2^ + 2*F*_c_^2^)/3
*S* = 1.08	(Δ/σ)_max_ = 0.002
1695 reflections	Δρ_max_ = 0.25 e Å^−^^3^
132 parameters	Δρ_min_ = −0.15 e Å^−^^3^
10 restraints	Absolute structure: Flack (1983), 745 Friedel pairs
Primary atom site location: structure-invariant direct methods	Flack parameter: 0.069 (10)

### Special details

**Table:**

Geometry. All e.s.d.'s (except the e.s.d. in the dihedral angle between two l.s. planes) are estimated using the full covariance matrix. The cell e.s.d.'s are taken into account individually in the estimation of e.s.d.'s in distances, angles and torsion angles; correlations between e.s.d.'s in cell parameters are only used when they are defined by crystal symmetry. An approximate (isotropic) treatment of cell e.s.d.'s is used for estimating e.s.d.'s involving l.s. planes.
Refinement. Refinement of *F*^2^ against ALL reflections. The weighted *R*-factor *wR* and goodness of fit *S* are based on *F*^2^, conventional *R*-factors *R* are based on *F*, with *F* set to zero for negative *F*^2^. The threshold expression of *F*^2^ > σ(*F*^2^) is used only for calculating *R*-factors(gt) *etc*. and is not relevant to the choice of reflections for refinement. *R*-factors based on *F*^2^ are statistically about twice as large as those based on *F*, and *R*- factors based on ALL data will be even larger.

### Fractional atomic coordinates and isotropic or equivalent isotropic displacement parameters (Å^2^)

**Table:**

	*x*	*y*	*z*	*U*_iso_*/*U*_eq_	
Ni1	0.15345 (3)	0.18215 (2)	0.53899 (3)	0.03234 (9)	
Cl2	0.03840 (8)	0.33531 (5)	0.61305 (6)	0.03892 (15)	
S2	0.33740 (7)	0.22386 (7)	0.41358 (6)	0.04019 (16)	
S1	−0.05478 (8)	0.10041 (6)	0.38067 (5)	0.03680 (15)	
Cl1	0.26641 (9)	0.06856 (6)	0.70409 (7)	0.04738 (16)	
C1	−0.1912 (3)	0.03547 (18)	0.4566 (2)	0.0312 (5)	
N1	−0.1424 (3)	0.0014 (2)	0.5748 (2)	0.0427 (5)	
N2	−0.3484 (3)	0.0180 (2)	0.3936 (2)	0.0419 (5)	
C2	0.5326 (3)	0.2585 (2)	0.5057 (2)	0.0341 (5)	
N3	0.6387 (3)	0.3127 (2)	0.4532 (3)	0.0535 (7)	
N4	0.5794 (3)	0.2294 (3)	0.6260 (2)	0.0562 (7)	
H1A	−0.042 (3)	−0.001 (3)	0.618 (3)	0.054 (10)*	
H2A	−0.412 (4)	−0.012 (3)	0.434 (3)	0.052 (9)*	
H3A	0.597 (8)	0.333 (4)	0.376 (3)	0.13 (2)*	
H4A	0.672 (3)	0.255 (3)	0.667 (3)	0.067 (11)*	
H1B	−0.217 (4)	−0.033 (3)	0.599 (3)	0.048 (9)*	
H2B	−0.392 (4)	0.048 (2)	0.327 (2)	0.039 (8)*	
H3B	0.737 (4)	0.327 (4)	0.501 (5)	0.095 (17)*	
H4B	0.513 (4)	0.186 (2)	0.656 (3)	0.046 (10)*	

### Atomic displacement parameters (Å^2^)

**Table:**

	*U*^11^	*U*^22^	*U*^33^	*U*^12^	*U*^13^	*U*^23^
Ni1	0.02675 (16)	0.03985 (16)	0.03008 (15)	−0.00044 (13)	0.00613 (11)	−0.00065 (13)
Cl2	0.0308 (3)	0.0446 (3)	0.0418 (3)	−0.0015 (2)	0.0094 (3)	−0.0120 (3)
S2	0.0226 (3)	0.0721 (4)	0.0250 (3)	−0.0055 (3)	0.0039 (2)	−0.0010 (3)
S1	0.0339 (3)	0.0500 (4)	0.0255 (3)	−0.0108 (3)	0.0052 (2)	−0.0026 (3)
Cl1	0.0441 (4)	0.0561 (4)	0.0385 (4)	0.0094 (3)	0.0032 (3)	0.0128 (3)
C1	0.0307 (12)	0.0278 (11)	0.0344 (13)	−0.0013 (9)	0.0063 (10)	−0.0036 (10)
N1	0.0366 (13)	0.0507 (13)	0.0385 (12)	−0.0128 (10)	0.0046 (10)	0.0096 (9)
N2	0.0301 (12)	0.0458 (14)	0.0462 (14)	−0.0054 (10)	0.0020 (10)	0.0098 (11)
C2	0.0230 (12)	0.0414 (13)	0.0366 (13)	0.0039 (10)	0.0045 (9)	−0.0070 (10)
N3	0.0270 (13)	0.0680 (18)	0.0648 (19)	−0.0072 (11)	0.0099 (13)	0.0030 (14)
N4	0.0326 (14)	0.095 (2)	0.0335 (13)	−0.0043 (14)	−0.0065 (11)	−0.0040 (14)

### Geometric parameters (Å, º)

**Table:**

Ni1—Cl1	2.2516 (7)	N1—H1B	0.822 (18)
Ni1—Cl2	2.2726 (7)	N2—H2A	0.840 (19)
Ni1—S2	2.3079 (7)	N2—H2B	0.806 (18)
Ni1—S1	2.3177 (6)	C2—N3	1.312 (4)
S2—C2	1.715 (2)	C2—N4	1.315 (4)
S1—C1	1.716 (2)	N3—H3A	0.87 (2)
C1—N1	1.312 (3)	N3—H3B	0.86 (2)
C1—N2	1.317 (3)	N4—H4A	0.836 (19)
N1—H1A	0.843 (19)	N4—H4B	0.865 (19)
			
Cl1—Ni1—Cl2	108.56 (3)	H1A—N1—H1B	120 (3)
Cl1—Ni1—S2	113.37 (3)	C1—N2—H2A	116 (3)
Cl2—Ni1—S2	114.86 (3)	C1—N2—H2B	124 (2)
Cl1—Ni1—S1	115.40 (3)	H2A—N2—H2B	117 (4)
Cl2—Ni1—S1	107.62 (3)	N3—C2—N4	119.7 (3)
S2—Ni1—S1	96.69 (2)	N3—C2—S2	118.7 (2)
C2—S2—Ni1	110.56 (9)	N4—C2—S2	121.6 (2)
C1—S1—Ni1	105.95 (8)	C2—N3—H3A	114 (4)
N1—C1—N2	119.3 (2)	C2—N3—H3B	117 (4)
N1—C1—S1	121.80 (19)	H3A—N3—H3B	128 (6)
N2—C1—S1	118.9 (2)	C2—N4—H4A	116 (3)
C1—N1—H1A	126 (3)	C2—N4—H4B	118 (3)
C1—N1—H1B	113 (3)	H4A—N4—H4B	126 (4)

### Hydrogen-bond geometry (Å, º)

**Table:**

*D*—H···*A*	*D*—H	H···*A*	*D*···*A*	*D*—H···*A*
N1—H1*A*···Cl1	0.84 (3)	2.60 (3)	3.388 (3)	157 (3)
N1—H1*B*···Cl2^i^	0.83 (3)	2.56 (3)	3.365 (3)	164 (3)
N2—H2*A*···Cl2^i^	0.83 (3)	2.75 (3)	3.499 (2)	150 (3)
N2—H2*B*···Cl2^ii^	0.81 (2)	2.64 (2)	3.432 (2)	166 (3)
N3—H3*A*···Cl1^iii^	0.86 (3)	2.83 (5)	3.423 (3)	128 (5)
N3—H3*B*···Cl2^iv^	0.86 (4)	2.47 (4)	3.317 (3)	168 (4)
N4—H4*A*···S2^v^	0.84 (3)	2.70 (3)	3.366 (2)	137 (3)
N4—H4*B*···Cl1	0.86 (3)	2.60 (3)	3.448 (3)	168 (3)

Symmetry codes: (i) *x*−1/2, *y*−1/2, *z*; (ii) *x*−1/2, −*y*+1/2, *z*−1/2; (iii) *x*+1/2, −*y*+1/2, *z*−1/2; (iv) *x*+1, *y*, *z*; (v) *x*+1/2, −*y*+1/2, *z*+1/2.
